# Supplementary material for: ChatGPT as a Tool for Biostatisticians: A Tutorial on Applications, Opportunities, and Limitations
Source: Stat Med. 2025 Oct 23;44(23-24):e70263. doi: 10.1002/sim.70263 (PMC12548020; doi:10.1002/sim.70263)
Supplement: Supplementary file 1 — Data S1. Supporting Information. [file SIM-44-0-s001.zip › Supplementary Materials Additional.zip!/3.1_Systematic_Reviews_and_Meta_aspects/3.1_Data Extraction - Base Prompt o4-mini - Chat 3.pdf]

Use Case Meta-Analysis - Data Extraction - Base Prompt o4-mini - Chat 3

Rudloff et al. - 2009 - Concurrent lobular neoplasia increases the risk of ipsilateral breast cancer recurrence in situ treated with breast-conserving therapy.pdf
PDF

Vidali et al. - 2012 - Conservative treatment of breast ductal carcinoma in situ results of an Italian multi-institutional.pdf
PDF

The aim is to perform a meta-analysis on total ipsilateral local recurrence rates at 10 years in cases of ductal carcinoma in situ

Data inclusion rules are:
To ensure the validity of the follow-up data, the results should be as close to 10 years follow-up (minimum) as the information in each study allows. If there is more than one publication from an institution or group, the most recent study with appropriate follow-up will be used to extract 10-year data.

Please provide:

- the rate of ipsilateral local recurrence for conservative surgery with additional radiotherapy (no other interventions or drugs allowed) after 10 years
- the number of eligible patients
- period of data collection
- duration of follow-up
- type of study
- age of patients (mean or median, maximum and minimum)
- treatment modalities

(Please provide a dataframe with one entry for each study, and column names "study", "rate", "n_patients", "period", "follow_up", "study_type", "age", and "treatment". Do not provide additional text).

```
markdown

| study                  | rate              | n_patients | period                            |
follow_up                          | study_type                          | age
| treatment
|
|----------------------|------------------|------------|---------------------------------|------
--------------------------------|-----------------------------------|--------------------------
-----------|-------------------------------------------------------------------------------------
----------------|
```

```
| Vidali et al. 2012 :contentReference[oaicite:9]{index=9} | 9.6% at 10 years
:contentReference[oaicite:10]{index=10} | 586 :contentReference[oaicite:11]{index=11}  | Feb
1985 – Mar 2000 :contentReference[oaicite:12]{index=12} | Median 136 mo (16–292 mo)
:contentReference[oaicite:13]{index=13} | Multi-institutional retrospective
:contentReference[oaicite:14]{index=14} | Median 55 y (range 29–84)
:contentReference[oaicite:15]{index=15} | Breast-conserving surgery + whole-breast RT (50 Gy);
boost in 295 pts (50%) at median 10 Gy :contentReference[oaicite:16]{index=16} |
```

Quellen
